# Novel Word Learning: Event-Related Brain Potentials Reflect Pure Lexical and Task-Related Effects

**DOI:** 10.3389/fnhum.2019.00347

**Published:** 2019-10-15

**Authors:** Beatriz Bermúdez-Margaretto, David Beltrán, Fernando Cuetos, Alberto Domínguez

**Affiliations:** ^1^Centre for Cognition and Decision Making, Institute for Cognitive Neuroscience, National Research University Higher School of Economics, Moscow, Russia; ^2^Instituto Universitario de Neurociencia (IUNE), Tenerife, Spain; ^3^Facultad de Psicología, Universidad de La Laguna, Tenerife, Spain; ^4^Facultad de Psicología, Universidad de Oviedo, Oviedo, Spain

**Keywords:** novel word learning, lexical decision task, reading task, event-related brain potentials, N400

## Abstract

Previous research has pointed out that the combination of orthographic and semantic-associative training is a more advantageous strategy for the lexicalization of novel written word-forms than their single orthographic training. However, paradigms used previously involve explicit stimuli categorization (lexical decision), which likely influence word learning. In the present study, we used a more automatic task (silent reading) to determine the advantage of the associative training, by comparing the brain electrical signals elicited in combined (orthographic and semantic) and single (only orthographic) training conditions. In addition, the learning effect (in terms of similar neurophysiological activity between novel and known words) was also tested under a categorization paradigm, enabling determination of the possible influence of the training task in the lexicalization process. Results indicated that novel words repeatedly associated with meaningful cues showed a higher attenuation of N400 responses than those trained in the single orthographic condition, confirming the higher facilitation in the lexico-semantic processing of these stimuli, as a consequence of semantic associations. Moreover, only when the combined training was carried out in the reading task did novel words show similar N400 responses to those elicited by known words, suggesting the achievement of a similar lexical processing to known words. Crucially, when the training is carried out under a demanding task context (lexical decision), known words exhibited positive enhancement within the N400 time window, contributing to maintaining N400 differences with novel trained words and confounding the outcome of the learning. Such deflection—compatible with the modulation of the categorization-related P300 component—suggests that novel word learning could be influenced by the activation of categorization-related processes. Thus, the use of low-demand tasks arises as a more appropriate approach to study novel word learning, enabling the build-up process of mental representations, which probably depends on pure lexical and semantic factors rather than being guided by categorization demands.

## Introduction

The development of reading fluency, namely the ability to visually recognize words with an adequate level of accuracy and speed, is essential for correct performance in most of our daily-life activities and critical for academic and professional success. It is accepted that repeated visual experience with novel word-forms allows the reader to evolve from slow, effortful and inaccurate reading, characterized by serial letter-by-letter decoding to automated and skilled reading, in which words are recognized through direct, parallel processing (Share, 1995, 2008). Thus, after a novel word-form has been decoded several times through purely visual experience, a mental representation is built-up in the reader’s lexicon, enabling its reading using this whole-word visual strategy (Meyer and Felton, 1999; Coltheart et al., 2001). Therefore, this so-called lexicalization process is crucial for the acquisition of the direct visual recognition of words and, ultimately, for developing fluent reading. However, the specific training which enables the integration of novel word-forms into the reader’s lexicon is still under debate.

Some behavioral studies have claimed that the formation of lexical representations is possible after single orthographic training with novel written word-forms, involving just a handful of repeated visual exposures under meaningless conditions, namely in the absence of any association to a semantic reference. Thus, this training is characterized as meaningless and non-associative, in which novel written-word forms are briefly exposed to participants through a short number of visual presentations (ranging from 4 and 10, depending on the study). In particular, these studies obtained the reduction of the length effect between short and long novel word-forms (Ellis et al., 2009; Maloney et al., 2009; Kwok and Ellis, 2015; Kwok et al., 2017; Suárez-Coalla et al., 2016) or an interference effect in the categorization of known words (Bowers et al., 2005; Qiao and Forster, 2013). Both results are taken as indexes of the representation of the novel items in the reader’s lexicon. However, contrary arguments can also be found in the literature. For instance, it is argued that such an interference effect is not indicative of the complete lexicalization of these stimuli but of the storage of episodic memory traces for them, which interfere during the categorization of known words (Leach and Samuel, 2007). Accordingly, other studies have shown that only when both orthography and the meaning of novel words are trained, is it possible to observe lexical competition effects between these stimuli and known words, in terms of a reduction of the prime lexicality effect (Qiao et al., 2009; Qiao and Forster, 2013). Therefore, some authors conclude that orthographic training is not enough to ensure the lexicalization of novel word-forms, with effects denoting the acquisition of interfering-episodic memory traces rather than competing-lexical representations after this training.

Nevertheless, given the rapid and dynamic changes that occur in the linguistic system during novel word learning, other measures than those which are behavioral are required to evaluate this process correctly. Thus, magneto-and electroencephalography methodologies, able to track online-processing changes in brain activity, are probably much more sensitive to assess novel word learning and, particularly, the nature of the neurophysiological mechanisms underlying a specific training with the orthographic or both the orthographic and semantic features of novel words. Regarding the effect of single orthographic training, rather few MEG/EEG studies are focused on neural dynamics during the acquisition of novel surface word-forms, with substantial methodological differences and inconsistent findings among them (Bermúdez-Margaretto et al., 2015, 2018; Partanen et al., 2018). For instance, in a recent MEG study, Partanen et al. (2018) found that massive (~100 repetitions) and unattended, parafoveal exposure to novel written word-forms caused an increase in the early brain activity, at around 100 ms post-stimulus onset. This enhancement, found after only 15 min of exposure with novel words outside the focus of the reader’s attention, was considered indicative of the rapid and automatic formation of lexical traces for these stimuli. However, different results have been found under paradigms better resembling the attentive context in which novel written word-forms are usually encountered. Thus, recent EEG research has shown that single orthographic training with novel word-forms enables the formation of memory traces for these stimuli whose nature is probably episodic rather than lexical (Bermúdez-Margaretto et al., 2015), in agreement with some behavioral studies discussed above (Qiao et al., 2009; Qiao and Forster, 2013). Specifically, short (up to six repetitions) visual exposure to novel word-forms in a lexical decision task caused an increase in amplitude in the late positive component (LPC), an ERP component traditionally related to episodic memory processes and recollection of previously presented information from long-term memory (for a review see Rugg and Curran, 2007). Hence, this LPC effect was considered to index the codification and strengthening of episodic memory traces that follow the repeated exposures of these stimuli. Given no modulation in lexical or lexico-semantic related ERP components was found as a consequence of this orthographic training, it was hypothesized that probably both novel word orthography and meaning should be trained in order to better instantiate them as lexical items.

This hypothesis was tested in a second study (Bermúdez-Margaretto et al., 2018), where we conducted a similar lexical decision task in which short orthographic training with novel word-forms (again, six repetitions) was compared to the effect of training both the orthography and the meaning of the stimuli, simultaneously. Thus, novel written word-forms were repeatedly presented in a single orthographic training (namely, a meaningless training condition) or in a combined orthographic/semantic training condition, where novel word-forms were trained through semantic-associative picture-word exposures (namely, a meaningful training condition). Replicating our previous findings, novel word-forms trained in the meaningless, non-associative condition showed an LPC enhancement across repetitions, reflecting the activation of episodic memory process through single orthographic training. Interestingly, a higher facilitation in the lexico-semantic processing of novel words was found when these stimuli were presented in the meaningful, semantic-associative training, reflected in a higher decrease in the N400 amplitudes for these stimuli in comparison to those trained in the meaningless condition. The modulation of this ERP component, typically related to semantic processing (Kutas and Federmeier, 2011), was taken as an index of the association between novel word-forms and picture-concepts throughout the meaningful training, in line with previous studies training novel words under meaningful conditions (Perfetti et al., 2005; Mestres-Missé et al., 2007; Borovsky et al., 2010; Frishkoff et al., 2010; Batterink and Neville, 2011; Angwin et al., 2014; Bakker et al., 2015). Notably, this advantage of the combined orthographic/semantic training over the single orthographic training had not been observed before, given that no direct comparison between both trainings had been provided before. Therefore, this study confirmed the effect of semantic training going beyond the enhancement of episodic memory processes, enabling the lexico-semantic facilitation of novel word-forms and probably contributing to their lexicalization to a higher extent.

In the above studies, the task used to guarantee stimuli processing during training was the lexical decision task, in which the primary aim is to categorize the upcoming stimuli—both known and novel words—as lexical/non-lexical items. This task forces the discrimination between known and novel words and could thereby facilitate the learning of the novel word-forms. Moreover, the particular semantic-associative training carried out in this task could further influence the learning of these stimuli, given that the preceding picture enabled their prediction and response anticipation. Thus, since the efficient picture-stimulus association ensured the faster and accurate categorization of the stimuli, participants probably followed an associative strategy in order to successfully fulfill the task requirement, leading to a higher facilitation in the processing of these stimuli and consequently lower N400 amplitudes. Therefore, the particular task context in which the semantic-associative training was carried out probably facilitated the development of a strategic-based learning, which, on the other hand, might be only indirectly related to the formation of the novel word as a lexical item. In this regard, it is possible that the N400 effect found in that study was not only reflecting facilitation in the lexico-semantic processing of stimuli but also its categorization during the task. Indeed, perceptual discrimination processes carried out in order to accomplish task requirements (as in this particular task, stimuli categorization) can also be reflected in this time window, as is the case of the P300 component (Polich, 1985, 2004; Picton, 1992).

Semantic processes are, however, considered to be rather automatic, with the access to stimulus meaning occurring in the absence of specific strategy or intention from the reader, although they might be modulated by higher top-down factors, such as temporal attention or task demands (Kiefer, 2008). For instance, automaticity in meaning access is reflected in the masked semantic priming effect, where the target processing is facilitated by a semantically related prime even when it is perceived unconsciously—and hence automatically (Carr and Dagenbach, 1990; Neely, 2012). Brain electrical signals also reflect such automaticity in semantic processing, with reduced N400 amplitudes elicited by targets preceded by semantically related masked primes (Deacon et al., 2000; Kiefer, 2002). Thus, facilitation in the lexico-semantic processing of novel word-forms could occur even if meaningful associations are carried out in a task involving a more automatic processing of the stimuli, such as a simple reading task.

Reading, besides preventing possible facilitation in word learning caused by categorization, is significantly less demanding than lexical decision since it involves a much more automatic processing of stimuli. Although some attention-demanding processes occur during reading (such as inference making or comprehension monitoring when reading texts), many others are automatic (such as letter identification or lexico-semantic access), particularly if reading of isolated words is considered (Perfetti, 1985; Walczyk, 2000). Indeed, lexico-semantic processes are accessed during this automatic-driven processing task even in the absence of a particular response; this has been evidenced in several studies, with the modulation of N400 when reading words semantically incongruent with the preceding sentence context (Kutas and Hillyard, 1980; Kutas and Van Petten, 1988). Therefore, the semantic-associative training of novel word-forms could facilitate the lexico-semantic processing of these stimuli during a reading task, confirming the advantage of this training for word lexicalization in the absence of confounding categorization effects. Moreover, this task would result in a more appropriate context to study the acquisition of mental traces for novel word-forms, since no other processes beyond those specifically related to word lexicalization—grapheme-to-phoneme decoding—are involved. In this sense, the presence of an N400 effect even with the suppression of categorization demands could indicate the formation of lexico-semantic traces, non-dependent on these processes but probably reflecting pure associative learning as a consequence of the training.

Therefore, the main goal of the present study was to determine whether the advantage of the combined training, over the single orthographic training, could be replicated under a training task free of categorization-confounding responses (silent reading), indicating the effectiveness of the semantic-associative training in novel word learning, or whether such an advantage was a consequence of the specific categorization context of the task (lexical decision). With this purpose, the present study carried out the same training paradigm as implemented before (Bermúdez-Margaretto et al., 2015)—thus, comparing single orthographic vs. orthographic/semantic trainings—but in this case, a silent reading task was used as a training context, instead of a lexical decision task. Importantly, this task shares the same materials, procedure, features of the sampled participants, EEG equipment and preprocessing pipeline as in the previous lexical decision task (for details see “Materials and Methods” section), making both tasks methodologically comparable. In particular, two main questions were separately addressed in this study.

First, we aimed to determine whether the combination of both orthographic and semantic training with novel word-forms facilitates the lexical processing of these stimuli to a higher extent than the single orthographic training, by using a task context in which the learning of the stimuli is not influenced by categorization demands. To address this question, the effect of both training conditions was tested along the silent reading task, in a similar way as carried out in our previous lexical decision task. We hypothesized that, as found in our previous study using the lexical decision task (Bermúdez-Margaretto et al., 2015), novel word-forms trained in the meaningful, semantic-associative condition in the present reading task will show greater facilitation in their lexico-semantic processing than non-associated stimuli, reflected in higher attenuation of N400 amplitudes. This training effect would indicate that, even in a task in which stimuli are processed automatically, the combination of orthographic and semantic training results in a more advantageous approach for their learning than in the case of the single orthographic training, with the progressive acquisition of meaningful content through associations to picture-concepts.

Additionally, we considered to explore the impact of the meaningful, semantic-associative training on the lexicality effect, namely in the differences between trained novel word-forms and already known words. This lexicality effect was not tested in our previous lexical decision task since that study was mainly focused on disentangling the effect of training novel words in single orthographic and combined conditions. Therefore, testing the N400 lexicality effect in both task contexts would provide further evidence about the acquisition of memory traces for semantically trained stimuli. Indeed, this effect is thought to reflect differences between already lexicalized stimuli and those without mental representations (Forster and Chambers, 1973; Glushko, 1979). Accordingly, previous studies have concluded that the reduction or absence of the N400 lexicality effect after semantic training evidences the achievement of the lexico-semantic status for trained stimuli (Mestres-Missé et al., 2007; Batterink and Neville, 2011; Bakker et al., 2015). Then, to address whether the semantic-associative training would lead to similar lexico-semantic processing between novel and known words and if this would occur to a different extent across tasks, we evaluated the N400 lexicality effect at the end of the meaningful, semantic-associative training in both tasks, the present silent reading and the previous lexical decision. Lexical differences between known and novel word-forms—and hence, a higher N400 lexicality effect—were expected in the lexical decision rather than in the reading task despite the learning, given the forced discrimination between known and novel words. However, a better match between the processing of novel and known words was expected in reading, confirming the formation of lexical, non-categorization-guided memory traces for stimuli trained in this particular task.

## Materials and Methods

### Participants

A group of 25 undergraduate psychology students took part in the present silent reading task for course credits (23 females; mean age of 21.48; SD: 2.04). All of them were native Spanish speakers, had normal or correct-to-normal vision and were right-handed according to the Oldfield’s Handedness Inventory (Oldfield, 1971). No psychiatric or neurological disorder was disclosed by any participant. This research was approved by the Ethics Committee of the Psychology Department of the University of Oviedo. Before starting the experimental tasks, participants received pertinent information about the purpose of the study, the tasks, and their duration. Written informed consent was then received from participants.

### Materials

The present silent reading task used the same materials and design as implemented in the previous lexical decision task (see Bermúdez-Margaretto et al., 2015). Hence, the task was divided into six blocks and the same set of 448 stimuli was used. Sixty-four of these stimuli were novel written word-forms (4–7 letter pseudowords, namely meaningless stimuli observing the orthographic and phonotactic Spanish rules, i.e., *pasne*), repeatedly presented from the first to the sixth block of the task. The remaining 384 stimuli were known words (4–7 letter Spanish nouns, i.e., *barba*), presented in sets of 64 stimuli in each task block. Therefore, these stimuli were not repeated but a new set of known words was presented in each task block. The aim of this procedure was to evaluate the lexicality effect in a more natural way, comparing the processing of a stimulus that is new and repeatedly encountered by the reader—and hence becoming familiar—with the processing of a stimulus that is already known and non-repeated. In sum, both tasks were composed of six blocks, each of them containing 128 stimuli, half of them known and the other half novel word-forms.

Additionally, half of the stimuli (both known and novel word-forms) in each task were repeatedly associated with a known concept by means of the previous presentation of a picture of a known object (semantic-associative condition with combined orthographic/semantic training). The other half of the stimuli were preceded by the presentation of a hash mark (#) not related to a known meaning (non-associative condition with single orthographic training). More specifically, known words (nouns) were associated with the corresponding picture of a known object in association with their meaning, maintaining correspondence between concepts represented by pictures and words. Thus, different pictures were presented in association with known words across blocks, whose selection was based on the word’s meaning. Regarding novel words, these stimuli were always associated to the same cue (a picture of a known object or hash mark) across repetitions. For this purpose, another set of pictures of known objects was selected (note that, target words for pictures associated to known and to novel words were counterbalanced in their familiarity and imageability). Pictures of known objects were obtained from the Snodgrass and Vanderwart set of pictures (Snodgrass and Vanderwart, 1980) and both pictures and hash marks had similar appearance and dimensions (10 × 15 cm). Table 1 shows the matching of the experimental stimuli in the main lexical (familiarity, imageability) and sub-lexical (frequency of bigrams and first syllable, number orthographic neighbors) psycholinguistic variables by means of the BuscaPalabras database (Davis and Perea, 2005).

**Table 1:** Matching means of each psycholinguistic variable through known and novel words compared for the present study (the same materials were used in both tasks).

		Known words Block 1 (associative condition)	Known words Block 6 (associative condition)	Novel words (associative condition)	Non-novel words (associative condition)	Test	*p*-value
Sub-lexical variables	Bigram frequency (token type)	590.46 (295.84)	598.30 (309.62)	516.28 (262.79)	515.32 (225.54)	*F*_(3,125)_ = 0.87	0.45
	Number of orthographic neighbors	3.37 (4.36)	2.46 (3.07)	2.68 (3.71)	1.31 (2.05)	*F*_(3,125)_ = 2.02	0.11
	First syllable frequency	291.36 (248.36)	255.45 (208.82)	271.45 (274.02)	306.67 (224.30)	*F*_(3,125)_ = 0.27	0.84
Lexical variables	Imageability	5.46 (1.75)	6.15 (0.36)	5.52 (2.16)	-	*F*_(2,95)_ = 1.79	0.17
	Familiarity	5.55 (1.83)	6.09 (0.68)	5.37 (2.14)	-	*F*_(2,95)_ = 1.61	0.20

*Standard deviations are shown in brackets. Statistical contrasts confirmed no significant differences across compared conditions (all *post hoc* contrasts resulted in *p* > 0.05)*.

### Procedure

First, an electrode cap was mounted on the scalp of participants, in order to record their EEG activity during the silent reading task. Verbal instructions were given to participants before starting the reading task, namely to pay attention and silently read each stimulus presented on the screen. This procedure was similar to that carried out in the previous lexical decision task, in which an explicit categorization of the stimuli was required (for details see Bermúdez-Margaretto et al., 2015). The researcher emphasized that participants should avoid blinks and muscular movements during the task and encouraged them to take breaks after each task block in order to prevent artifacts and fatigue. Before starting the experiment, instructions for the task appeared on the computer screen followed by eight training trials.

**Figure 1:**
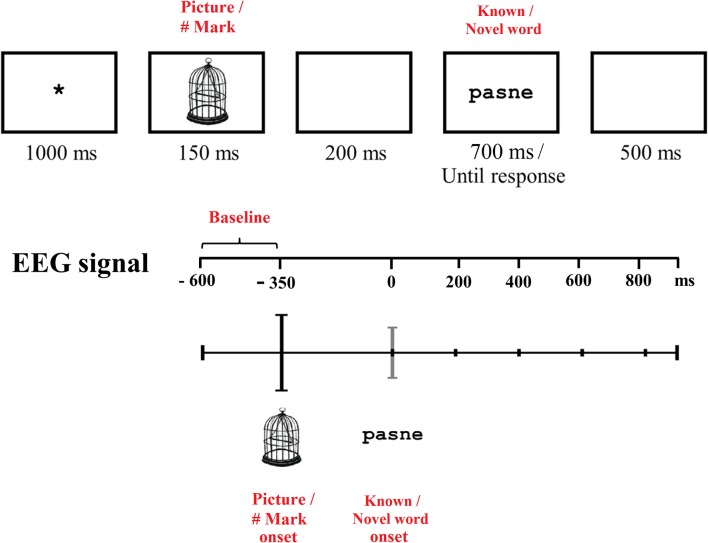
Sequence of stimuli presentation during both reading and lexical decision tasks. During the semantic-associative condition, a picture was presented in association with stimuli (known or novel word-forms, as depicted in the figure) whereas during the non-associative condition, a hash mark (#) was displayed. The latency of elements presented across the sequence is indicated by the numbers displayed at the bottom of each rectangle. Note that the target stimulus (a known word, i.e., *barba* or a novel word, i.e., *pasne*) was presented either for 700 ms (in the reading task) or until the participant responded (in the lexical decision task). The scale below represents the time (in milliseconds) corresponding to the EEG signal recording. The 250 ms preceding the picture/hash mark onset was used as a baseline.

Stimuli were displayed in black Verdana 18 point letters (known and novel words) or in black line drawings (pictures and hash marks) over a white background in the center of the screen by means of the E-Prime 2.0 software (Schneider et al., 2002). All trials were presented in randomized order within each task block. The sequence of stimuli presentation in the current reading task was identical to that of the lexical decision task employed by Bermúdez-Margaretto et al. (2015). In particular, the sequence started with a fixation cross displayed in the center of the screen for 1,000 ms. Then, a picture (for semantic-associative trials) or a hash mark (for non-associative trials) was presented for 150 ms, followed by a 200 ms blank screen. Afterward, the target (a known or a novel word) was presented on the screen for 700 ms (or until participant’s response, for the lexical decision task). Finally, another blank screen was presented for 500 ms; see Figure 1 for the sequence of stimuli presentation in both tasks.

### Recording and Pre-processing of the EEG Data

Brain electrical signals were recorded during the present reading task by means of an EEG equipment with 64 Ag/AgCl actiCAP electrodes (Brain Products GmbH, Gilching), similarly to that used in the previous lexical decision task, mounted in an elastic cap according to the 10/20 system (Jasper, 1958). The inter-electrode impedance of active electrodes was kept under 25 kΩ. Ocular activity was recorded by two electrodes placed on the infraorbital and supraorbital canthus of the left eye. The activity in both mastoid bones was also recorded to calculate an offline reference. During the online recordings, the EEG signal was referenced to the activity of the vertex electrode (Cz). The EEG and EOG signals were digitalized and amplified by an actiCHamp amplifier system (Brain Products GmbH, Gilching) at a 1,000 Hz sampling rate. A notch filter at 50 Hz was applied and 0.1 and 100 Hz high and low pass filters were set.

Pre-processing of EEG signals collected from the task was implemented using MATLAB software (The Mathworks Inc.) by using the Fieldtrip Toolbox (Oostenveld et al., 2011). The pre-processing steps were the same as those implemented in the previous lexical decision task. First, an artifact rejection was carried out in order to eliminate trials with amplitude values exceeding ±100 μV. Next, an independent component analysis (ICA) was run to detect and correct visual artifacts, and then a new artifact rejection was applied to ensure the total rejection of artifacts in data. The signal was segmented in periods of 1,500 ms, from −600 to 900 ms post target onset (from −600 to 1,000 ms post target onset for the lexical decision task). The baseline was corrected using the 250 ms preceding the picture/hash mark onset. A new reference was calculated using the mean activity of the mastoid electrodes, applied to 62 electrodes with the activity of the online reference (Cz) recovered. A new sampling rate was established at 256 Hz and a low pass band filter was applied at 30 Hz. ERPs were computed by averaging segments per subject and per condition.

### ERP Data Analysis

**Figure 2:**
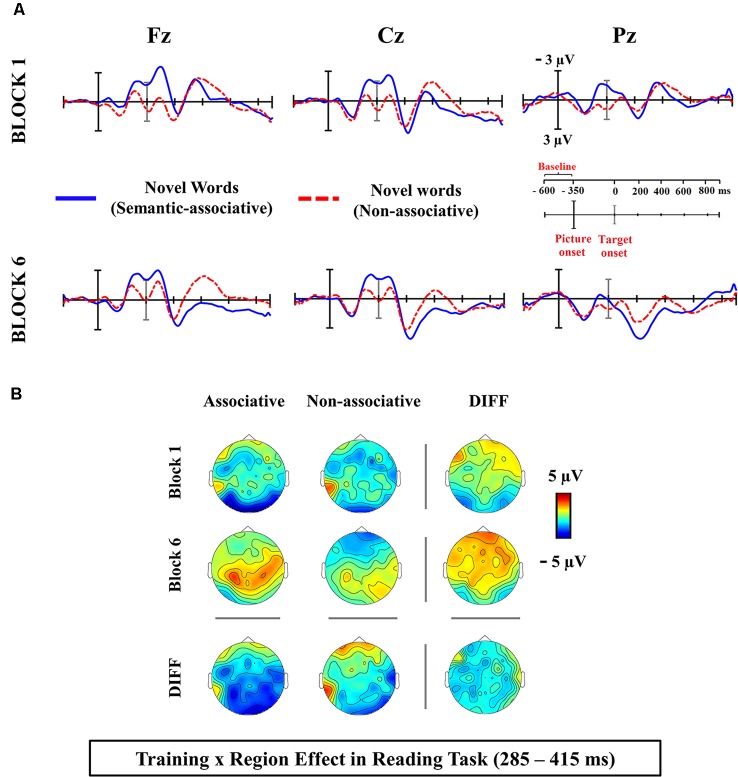
**(A)** Averaged ERP waveforms at electrodes from the medial scalp sites for novel word-forms at the semantic-associative and non-associative training conditions in the first and sixth block of the silent reading task. The interactive training × region effect confirmed that semantically-associated novel word-forms exhibited significantly less negative N400 amplitudes than those repeated under the simple, non-associative condition and, particularly, at frontal and central scalp regions. **(B)** Topographical maps showing the ERP activity for each condition. Maps under or to the right of the DIFF label show the scalp distribution of the differences between conditions.

**Figure 3:**
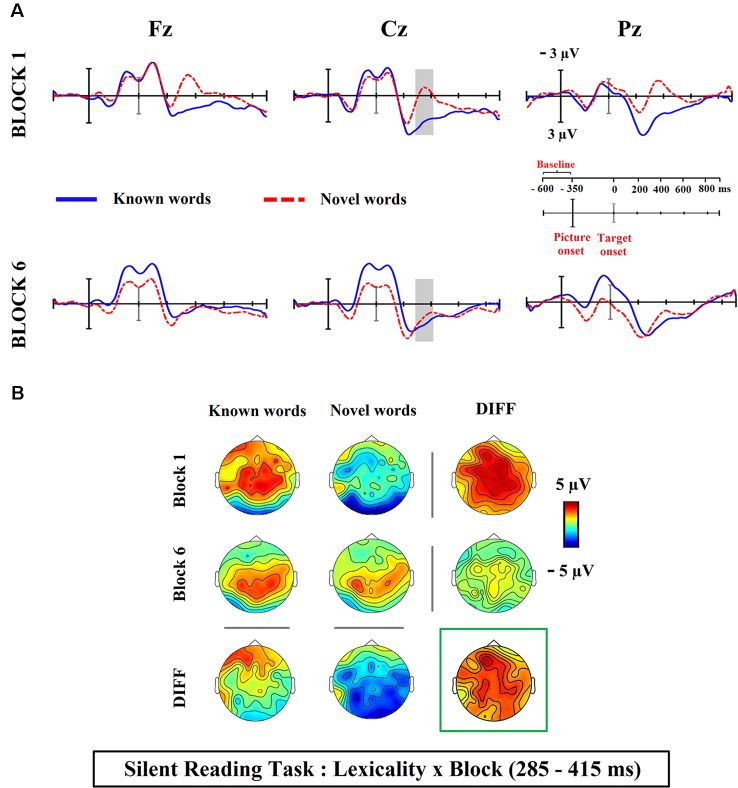
**(A)** Averaged ERP waveforms at electrodes from the medial scalp sites for known and novel word-forms after their semantic-associative training at the silent reading task. Gray shaded area reflects the time window of the significant interaction between lexicality and block. Follow up comparisons revealed that initial differences between known and novel word-forms were eliminated at the end of the task, as a consequence of the semantic-associative training. **(B)** Topographical maps showing the ERP activity for each condition. Maps under or to the right of the DIFF label show the scalp distribution of the differences between conditions. The map framed in green represents the scalp distribution of the interactive effect. In this task only novel word-forms modulated the N400 component, as indicated by the morphology of waveforms along with topographical maps, with no positive deflection at posterior sites compatible with the modulation of the P300 component.

**Figure 4:**
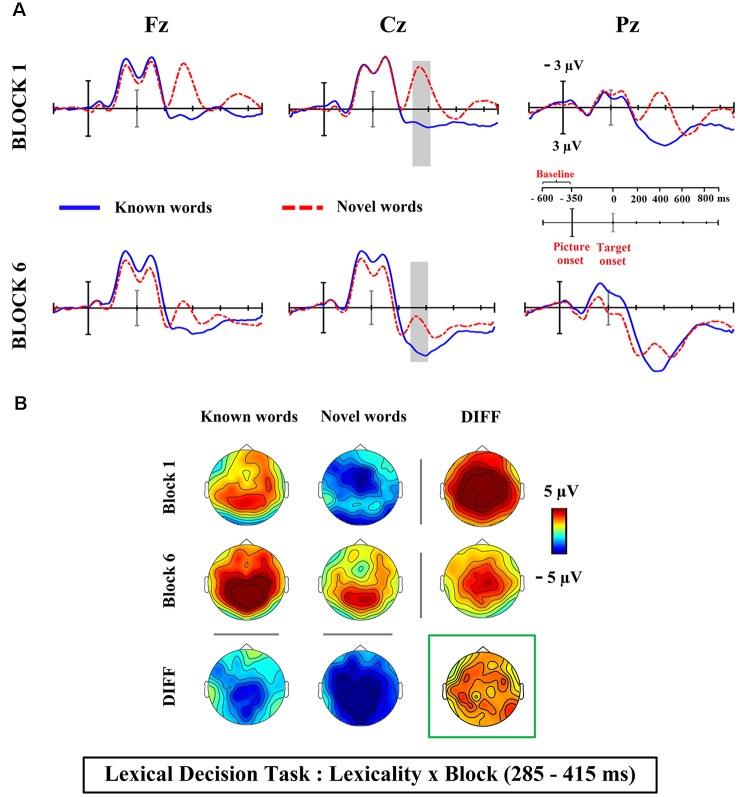
**(A)** Averaged ERP waveforms at electrodes from the medial scalp sites for known and novel word-forms after their semantic-associative training at the lexical decision task. Gray shaded area reflects the time window of the significant interaction between lexicality and block. Follow-up comparisons confirmed differences in the N400 amplitude between known and novel word-forms were reduced but still found significant at the end of the associative training. **(B)** Topographical maps showing the ERP activity for each condition. Maps under or to the right of the DIFF label show the scalp distribution of the differences between conditions. The map framed in green represents the scalp distribution of the interactive effect. Note that, whereas repetition of novel word-forms modulated the N400 component (negative deflection maximal at frontal and central scalp sites), the presentation of known words caused a positive deflection maximal at posterior scalp sites; this positive deflection is not observed in the reading task, where only novel word-forms modulated the N400 component. Both the morphology of waveforms and topographical maps suggest this positive enhancement is probably compatible with the modulation of P300, with the overlap of both N400 and P300 components during lexical decisions.

Visual inspection of ERP waveforms obtained at the present silent reading task revealed a reduction (from first vs. sixth block) in the amplitude of novel word-forms trained under the associative condition, in comparison to those presented under the single orthographic training. Such training effect reached maximum around 300 ms post-stimulus onset at frontal and central scalp electrodes, likely reflecting the different influence of both training conditions in the N400 component (see Figure 2). The inspection of the ERP waveforms for the lexicality effect (differences between novel and known word-forms trained in associative condition) also showed a modulation in the N400 latency, for both silent reading and lexical decision tasks (see Figures 3, 4). Then, for each task, a temporal window from 285 to 415 ms was selected and the mean activity of known and novel word-forms after the training was extracted in representative midline electrodes (AFZ/3/4, CZ/1/2 and POZ/3/4), where the ERP component of interest (N400) usually peaks at central sites. Two different analyses were carried out to address our hypotheses.

First, we aimed to determine whether the semantic-associative training caused higher facilitation in the lexico-semantic processing of novel word-forms than the non-associative training in a task that was context free of categorization-confounding demands (namely, in the reading task). For this purpose, the effect of the associative and the non-associative conditions was evaluated through the present silent reading task by means of a 2 × 2 × 3 repeated measures ANOVA with training (associative and non-associative), block (first and sixth) and region (frontal, central and posterior) as within-subject factors.

Second, we aimed to further analyze the impact of the associative training in the lexicality effect (namely, in the differences between known and novel word-forms before and after their associative training) in the present reading task as well as at the previous lexical decision task. Thus, a 2 × 2 × 3 repeated measures ANOVA with lexicality (known and novel word-forms), block (first and sixth) and region (frontal, central and posterior) as within-subject factors, was computed separately for the silent reading and the lexical decision tasks.

## Results

### Effect of Training in Silent Reading Task

The 2 × 2 × 3 repeated measures ANOVA with the type of training (novel word-forms after associative and non-associative training), block (first and sixth) and region (frontal, central and posterior) conducted for the reading task revealed main effects of block (*F*_(1,24)_ = 7.031, *p* = 0.014, $\eta_{\text{p}}^{2}$ = 0.22, 1-*β* = 0.72) and region (*F*_(2,48)_ = 4.32, *p* = 0.019, $\eta_{\text{p}}^{2}$= 0.15, 1-*β* = 0.72), as well as significant interactions between training and region (*F*_(2,48)_ = 4.11, *p* = 0.022, $\eta_{\text{p}}^{2}$ = 0.14, 1-*β* = 0.70) and block × region (*F*_(2,48)_ = 5.91, *ε* = 0.77, $\eta_{\text{p}}^{2}$ = 0.19, 1-*β* = 0.78). No other effects or interactions reached significance (*p* > 0.05). The training × region interaction was tested again in a separate ANOVA collapsing the two levels of the factor block (*F*_(2,48)_ = 4.11, *p* = 0.022, $\eta_{\text{p}}^{2}$ = 0.14, 1-*β* = 0.70). Follow-up comparisons for the effect of training in each scalp region revealed that differences between training conditions were frontally distributed (see topographic maps in Figure 2); thus, novel word-forms repeated under the associative training condition exhibited significantly less negative N400 amplitude than those under the non-associative training condition at frontal (*F*_(1,24)_ = 9.38, *p* = 0.005, $\eta_{\text{p}}^{2}$ = 0.28, 1-*β* = 0.83; semantic-associative: −0.69 μV, non-associative: −1.91 μV) and, marginally, at central regions (*F*_(1,24)_ = 3.19, *p* = 0.08, $\eta_{\text{p}}^{2}$ = 0.11, 1-*β* = 0.40), but not at posterior scalp sites (*F*_(1,24)_ = 0.015, *p* = 0.90, $\eta_{\text{p}}^{2}$ = 0.001, 1-*β* = 0.05; see Figure 2). Hence the repeated exposure to novel word-forms under the combination of orthographic and semantic training resulted in less negative N400 responses than under the simple visual condition, and irrespectively on the task block. Nonetheless, the interaction training × block, although marginal (*F*_(1,24)_ = 3.48, *p* = 0.07, $\eta_{\text{p}}^{2}$ = 0.12, 1-*β* = 0.43), suggests that both training conditions changed differently across blocks, with higher N400 reduction exhibited by semantically associated novel word-forms across blocks (diff.: −2.23 μV) than those repeated under the non-associative training condition (diff.: −0.83 μV); which in turn increased differences between training conditions, from the first (diff.: 0.01 μV) to the last task block (diff.: 1.40 μV).

Therefore, in agreement with previous findings using a lexical decision task as training context, the combination of both orthographic and semantic trainings caused a higher reduction of N400 amplitudes elicited by novel word-forms than the simple non-semantic training condition. Importantly, such advantage for the semantic-associative training over the non-associative training was found in the present study in a task free of categorization demands and wherein stimuli are processed automatically.

### Changes in Lexicality Effect in the Reading and in the Lexical Decision Tasks

The 2 × 2 × 3 repeated measures ANOVA carried out for the silent reading task with lexicality (known and novel word-forms after semantic-associative training), block (first and sixth) and region (frontal, central and posterior) revealed the main effects of lexicality (*F*_(1,24)_ = 10.57, *p* = 0.003, $\eta_{\text{p}}^{2}$ = 0.30, 1-*β* = 0.87) and region (*F*_(2,48)_ = 4.86, *p* = 0.012, $\eta_{\text{p}}^{2}$ = 0.16, 1-*β* = 0.77), as well as lexicality × block (*F*_(1,24)_ = 11.93, *p* = 0.002, $\eta_{\text{p}}^{2}$ = 0.33, 1-*β* = 0.91) and block × region (*F*_(2,48)_ = 8.49, *p* = 0.001, $\eta_{\text{p}}^{2}$ = 0.26, 1-*β* = 0.95) interactions. No other effects were found significant (*p* > 0.05). The lexicality × block interaction was tested again in a separate ANOVA collapsing the three levels of the factor region (*F*_(1,24)_ = 12.03, *p* = 0.002, $\eta_{\text{p}}^{2}$ = 0.33, 1-*β* = 0.91). Follow-up comparisons revealed that differences between novel and known word-forms in the first block (*F*_(1,24)_ = 19.64, *p* = 0.000, $\eta_{\text{p}}^{2}$ = 0.45, 1-*β* = 0.98, diff.: 3.45 μV) were eliminated at the end of the training at the sixth block (*F*_(1,24)_ = 0.58, *p* = 0.45, $\eta_{\text{p}}^{2}$ = 0.024, 1-*β* = 0.11; diff.: 0.46 μV). Thus, the repetition of novel word-forms at the semantic-associative condition caused a significant modulation in their N400 amplitude across task blocks (*F*_(1,24)_ = 9.63, *p* = 0.005, $\eta_{\text{p}}^{2}$ = 0.28, 1-*β* = 0.84, first block: −1.33 μV, sixth block: 0.89 μV), an effect which was not found for known words (*F*_(1,24)_ = 0.81, *p* = 0.37, $\eta_{\text{p}}^{2}$ = 0.033, 1-*β* = 0.14, first block: 2.11 μV, sixth block: 1.36 μV; see Figure 3).

The data set of the lexical decision task (Bermúdez-Margaretto et al., 2015) was submitted to the same 2 × 2 × 3 repeated measures ANOVA, showing significant main effects of lexicality (*F*_(1,21)_ = 51.80, *p* = 0.000, $\eta_{\text{p}}^{2}$ = 0.71, 1-*β* = 1) and block (*F*_(1,21)_ = 31.39, *p* = 0.000, $\eta_{\text{p}}^{2}$ = 0.59, 1-*β* = 1), as well as lexicality × block (*F*_(1,21)_ = 4.12, *p* = 0.05, $\eta_{\text{p}}^{2}$ = 0.16, 1-*β* = 0.49), lexicality × region (*F*_(2,42)_ = 6.07, *ε* = 0.77, $\eta_{\text{p}}^{2}$ = 0.22, 1-*β* = 0.79) and block × region (*F*_(2,42)_ = 6.37, *p* = 0.004, $\eta_{\text{p}}^{2}$ = 0.23, 1-*β* = 0.87) interactions. The lexicality × block interaction was tested again in a separated ANOVA collapsing all three levels of the factor region (*F*_(1,21)_ = 4.12, *p* = 0.05, $\eta_{\text{p}}^{2}$ = 0.16, 1-*β* = 0.49). Contrary to results obtained in the silent reading task, follow-up analysis revealed that differences between novel and known words found at the beginning of the semantic-associative training (*F*_(1,21)_ = 71.86, *p* = 0.000, $\eta_{\text{p}}^{2}$ = 0.77, 1-*β* = 1; diff.: 4.87 μV) were reduced but still remained significant at the last block of the training (*F*_(1,21)_ = 9.12, *p* = 0.006, $\eta_{\text{p}}^{2}$ = 0.30, 1-*β* = 0.82; diff.: 2.70 μV, see Figure 4). Interestingly, the N400 amplitude resulted as modulated across the lexical decision task not only for novel word-forms (*F*_(1,21)_ = 22.67, *p* = 0.000, $\eta_{\text{p}}^{2}$ = 0.51, 1-*β* = 0.99; first block: −2.80 μV, sixth block: 2.09 μV) but also for known words (*F*_(1,21)_ = 16.77, *p* = 0.001, $\eta_{\text{p}}^{2}$ = 0.44, 1-*β* = 0.97; first block: 2.07 μV, sixth block: 4.79 μV). Indeed, known words in the lexical decision task elicited a positive modulation in the N400 time window which was absent in the reading task, as can be observed in the ERP waveforms for known words displayed in Figure 4. Such positivity could be compatible with the modulation of the P300 component, with both P300 and N400 components overlapping at the same latency.

Therefore, the semantic-associative training resulted in a different modulation of the N400 lexicality effect at both tasks, with the elimination of differences between known and novel word-forms in the reading task but not in the lexical decision task (although no differences were found between known and novel word forms in reaction times or errors after their semantic-associative training in the lexical decision task, see Supplementary Material). However, the positive enhancement elicited by known words in the lexical decision task probably contributed to maintaining lexical differences; indeed, lexicality was found eliminated in the reading task, where such positivity was not enhanced.

## Discussion

The present study aimed to determine whether task demands modulate previously reported advantage for novel word lexicalization in the combination of orthographic and semantic-associative training, as compared to single orthographic training. More specifically, we evaluated the impact of these two different training conditions under a more automatic task than that used before for this purpose (lexical decision task), in which an explicit stimuli categorization was required. Thus, we first tested the impact of both types of training on the N400 amplitude through a task free of categorization demands (namely, a silent reading task), in a similar way as previously carried out using a lexical decision task as a training context (Bermúdez-Margaretto et al., 2018). Second, we evaluated the differences in brain activity between newly trained and already known word-forms (i.e., lexicality effect) at both task contexts, as more conclusive proof for the build-up of mental traces into reader’s lexicon as a consequence of the training. The results in the present silent reading task confirmed the stronger facilitation in the lexico-semantic processing of novel word-forms after their semantic-associative training in comparison to their single, orthographic training—as reflected in longer reduction of N400 amplitudes for the associative than for the non-associative condition. However, despite the fact that N400 training effect was obtained in both tasks, only those novel word-forms trained in the silent reading task reached a similar lexico-semantic processing to known, already lexicalized words. In contrast, for the lexical decision task, novel words remained showing larger N400 amplitudes than known words after the training, which could be explained by a possible overlap between lexico-semantic and categorization processes, particularly evident for known words. In what follows, ERP findings from both analyses, as well as their implications for novel word learning, are discussed in detail.

The brief exposure to novel written-word forms in association with meaningful cues resulted in the modulation of the N400 amplitude. Similar findings, indicative of the facilitation in the lexico-semantic processing of novel words, have been reported in prior research after the repetition of these stimuli in association to pictures (Dobel et al., 2010; Angwin et al., 2014; Bermúdez-Margaretto et al., 2018) and definitions (Perfetti et al., 2005; Bakker et al., 2015) or embedding them in meaningful sentence contexts (Mestres-Missé et al., 2007; Borovsky et al., 2010; Frishkoff et al., 2010; Batterink and Neville, 2011). Interestingly, recent research has provided a specific comparison between this meaningful exposition and the single orthographic training of novel words (visual repetition), disentangling both effects and highlighting the advantage of the combined orthographic and semantic training for novel word learning (Bermúdez-Margaretto et al., 2018). In this sense, when both novel word’s orthography and meaning were simultaneously trained, higher impact was found in their lexical processing as evidenced in lower N400 amplitudes; in contrast, single orthographic training mainly influenced the episodic processing of these stimuli, as reflected in the LPC enhancement across repeated visual exposures. Nonetheless, a potential confound between lexicalization and categorization processes must be noted in this research, since demands in this task (lexical decisions) could lead to higher discrimination and learning of the stimuli and, importantly, to the acquisition of categorization-guided rather than pure lexical representations for trained word-forms. However, results obtained in the present reading task confirm that, in the absence of such categorization response which could facilitate the learning, a training effect was also obtained, with lower negative N400 amplitudes after combined training. Thus, even in a task without categorization demands and, hence, reflecting likely automatic, and superficial processing of trained stimuli, the passive exposure across meaningful associations leads to a deeper influence in their lexico-semantic instantiation. Therefore, this finding suggests the combination of both orthographic and semantic-associative training could result in a more advantageous strategy for the integration of novel written-word forms into the linguistic system of readers, supporting previous statements. Furthermore, it shows that novel word learning processes can be rather automatic, with the lexico-semantic processing of stimuli accessed and modulated even during a task in which no response is required from readers. Moreover, these findings extend previous results found in this strand of research, which have shown the rapid and automatic acquisition of memory traces for novel written word-forms after their fully unattended, parafoveal exposure (Partanen et al., 2018). In this sense, word learning effects have been found even when reader’s attention is directed to different stimuli.

Nonetheless, although such advantage for the associative training is found at both the present silent reading task and at the previous lexical decision task (Bermúdez-Margaretto et al., 2018), the level of automaticity seems to differ between both task contexts, which likely leads to a difference in processing of novel words along their training and hence to their different learning. Indeed, the influence of the task was evident when we evaluated the impact of the meaningful training in the achievement of trained novel words as lexical entities—measured in the N400 lexicality effect. Whereas N400 differences between novel and known words resulted as being eliminated after the meaningful training in the reading task, the N400 lexicality effect remained significant in the lexical decision task. Such differential N400 lexicality effect is probably a consequence of the influence of categorization processes in this specific task, as particularly evidenced in the brain activity exhibited by known words. In this sense, these stimuli elicited a positive enhancement within the N400 time window; taking into account the positive polarity and more posterior topographical distribution of this effect, the processing of known words being likely to affect the P300 component, with the simultaneous modulation of both N400 and P300 peaks during this task. This component, related to attentional mechanisms activated to accomplish task requirements, such as stimuli categorization (Polich, 1985, 2004; Picton, 1992), is probably reflecting the reader’s strategy about the incoming stimuli, addressed to carry out the efficient stimuli categorization during the lexical decision task. Remarkably, such P300 deflection was observable for known but not for novel word-forms, probably contributing to maintaining lexical differences between both stimuli. In this sense, it is possible that the lexico-semantic processing of known words—and consequently the modulation of N400—could be less influenced than for novel words, regardless of their association to meaningful cues. This would lead to a highly evident P300 deflection in the ERP waveforms for known words. In contrast, for novel word-forms, the modulation of the N400 elicited by their repeated association to meaningful cues probably overlaps the activity of the P300 component.

An alternative explanation must also be taken into account; it is also possible that repetition of novel word-forms leads to a lower modulation of P300 for these stimuli than for known words, which were not trained across the task. To further explore this question, future studies should consider the training of known words, as this control could clarify whether lower P300 modulation for trained novel words is a consequence of a decrease in stimuli attention driven by their repeated exposure. Nonetheless, since novel word-forms were required to be categorized, it is rather possible that their lexico-semantic processing was also influenced by the activation of categorization-related processes during learning, as occurred for non-repeated known words, and hence confounding the outcome of the learning. Besides this, other limitations of the present research should be taken into account in future studies by evaluating not only electrophysiological but also behavioral outcomes for the learning of novel words (as well as for general reading abilities in both experimental groups), and testing this process in greater samples than those tested in the tasks reported in this study.

On the other hand, when the task in which the training is carried out does not require a specific categorization response, leading to a more shallow discrimination of the stimuli, no modulation of P300 is observed even in the case of known words. Therefore, the lexico-semantic processing of novel word-forms trained in the reading task was probably not confounded by categorization demands, enabling the construction of mental representations which depend on purely lexical and semantic factors rather than guided by categorization demands. Moreover, when compared to known words, the lexico-semantic processing of both stimuli is matched, as no categorization was required in this task which could cause the modulation of P300 activity for words, leading to lexical differences between these stimuli and novel words.

Therefore, the present study suggests a probable co-occurrence of both N400 and P300 components in the lexical decision task, reflecting the temporal overlap between different cognitive processes, namely, semantic-associative and task-related, categorization processes. The possible overlap between both components has been discussed in the electrophysiological literature, highlighting that effects attributed to N400 modulations could in fact being caused by an underlying P300 modulation (Rugg, 1990). However, not many studies have empirically explored this ERP co-occurrence in the lexico-semantic domain. For instance, in Roehm et al. (2007), a P300 component was found to be modulated depending on the task, with overlap between this component and N400 when the target was highly predictable and also relevant to solve the tasks. Hence, the P300 deflection observed in the present study is consistent with these previous findings and suggests that the P300 modulation is contributing to the lexicality effect obtained in the lexical decision task.

As claimed in Roehm et al. (2007), effects initially attributed to N400 can actually be influenced by P300 modulations (Bentin, 1987; Kutas and Iragui, 1998; Federmeier and Kutas, 1999). In this regard, the interpretation of the differential lexicality effect found in both tasks should be cautiously addressed, taking into account the simultaneous co-occurrence of both ERP effects. Thus, the remaining N400 lexicality effect is not reflecting the poor lexico-semantic learning of novel word-forms in the lexical decision task; on the contrary, the strong modulation of the N400 amplitude along the task proved the facilitation in the processing of these stimuli. Contrarily, lexical differences are probably maintained as a consequence of the P300 modulation elicited by the categorization of known words. Altogether, these findings suggest that N400 modulations found in language learning paradigms must be carefully explored, considering the possible P300 modulations that can occur simultaneously within the N400 time window as a consequence of task-related strategies. Given the potential confounding between both effects, cautious conclusions about the processes under study must be provided.

In short, the present study confirms the advantage in the processing of novel written words, as a consequence of their semantic-associative repetition, by using a silent reading task free of categorization-confounding demands. Thus, this associative training was found to cause a stronger N400 modulation than the single orthographic exposure even under a low-level demand task, which likely induced the lexicalization of these stimuli as suggested by the elimination of the N400 lexicality effect. Importantly, the brain activity for novel and known word-forms was not found matched when the training was carried out under a lexical decision task. Such differential lexicality effect found across both tasks probably suggests the different influence of each task context in the build-up process of mental representations for novel word-forms: purely related to lexico-semantic processes in the reading task or possibly confounded by categorization processes in lexical decision. Therefore, this pattern of results indicates the higher suitability of the reading task over the lexical decision task to study the associative learning of novel words in the absence of confounding categorization processes. In this sense, a final remark should be provided regarding the specific task used to address novel word learning. The present study shows that lexico-semantic learning can be effectively studied by using low-level demand tasks, in which no particular response is required from readers. Thus, tasks demanding particular responses and involving higher discrimination of the stimuli, such as lexical decision, should be used with caution to study the lexicalization of novel word-forms, since they introduce processes which are probably not involved during word learning.

## Data Availability Statement

The datasets generated for this study are available on request to the corresponding author.

## Ethics Statement

This research was approved by the Ethics Committee of the Psychology Department of the University of Oviedo. Before starting the experimental tasks, participants received pertinent information about the purpose of the study, the task, and their duration. Then, written informed consent was received from participants.

## Author Contributions

BB-M conducted the experimental tasks and analyzed the data. BB-M and DB wrote the manuscript. AD and FC designed the experimental tasks.

## Conflict of Interest

The authors declare that the research was conducted in the absence of any commercial or financial relationships that could be construed as a potential conflict of interest. The reviewer MV declared a shared affiliation, though no other collaboration, with several of the authors (DB, AD) to the handling Editor.
