# Supplementary material for: Novel Word Learning: Event-Related Brain Potentials Reflect Pure Lexical and Task-Related Effects
Source: Front Hum Neurosci. 2019 Oct 15;13:347. doi: 10.3389/fnhum.2019.00347 (PMC6803495; doi:10.3389/fnhum.2019.00347)
Supplement: Supplementary file 1 [file Data_Sheet_1.docx]

**Supplementary material**

**Behavioral data analysis**

Mean reaction times and errors obtained in the lexical decision task are presented in the Supplementary Table. ANOVAs across both participants (F1) and across items (F2) were carried on latencies and number of errors. Following same analysis approach as conducted in ERP data, both latency and accuracy indexes were compared between pseudowords trained under semantic-associative and non-associative conditions, in order to test whether behavioral outcomes differed depending on the training carried out. Additionally, in order to determine whether the meaningful, semantic associative training caused the elimination of lexical differences (namely, between novel and known, already lexicalized words), reaction times and errors for novel and known words were compared at the end of associative training

The analysis carried out on the latencies showed differences in RTs for novel words after semantic-associative and non-associative trainings both in analysis by participants (*F*1_1,21_=102.47, *p*=.000, *η*_p_²=.83, 1-*β*=1) and by items (*F*2_1,64_=325.86, *p*=.000, *η*_p_²=.84, 1-*β*=1). In particular, lower RTs were found for novel words trained in association to a picture than those trained under the non-associative condition (459.03 vs 574.70 ms, respectively). Indeed, at the end of the associative-semantic training, the latencies obtained for novel words were found similar to those exhibited by known words, although only in the analysis by participants (*F*1_1, 21_= 3.28, *p*=.084, *η*_p_²=.13, 1-*β*=.40; *F*2_1,64_=8.89, *p*=.004, *η*_p_²=.12, 1-*β*=.83, 459.030 vs. 436.39 ms, respectively).

Regarding accuracy data, a general ceiling effect was found in the performance of the lexical decision task, with the percentage of correctly responded stimuli above 90%. The analysis on errors revealed differences between the number of errors committed for novel words at the end of both semantic-associative and non-associative, although only reaching significance in the analysis by items (*F*1_1,21_=3.65, *p*=.07, *η*_p_²=.14, 1-*β*=.44; *F*2_1,64_=4.91, *p*=.03, *η*_p_²=.073, 1-*β*=.58). Thus, lower mean of error was found for those novel words trained under the meaningful, associative training than for those under the meaningless, single orthographic training (0.45 vs 1.13 mean of error, respectively). Furthermore, the comparison with known words showed similar accuracy between novel and known words after the semantic-associative training (*F*1_1, 21_= .074, *p*=.78, *η*_p_²=.003, 1-*β*=.058; *F*2_1,64_=.056, *p*=.81, *η*_p_²=.001, 1-*β*=.56, 0.45 vs. 0.40 mean of error, respectively).

Supplementary Table. Mean reaction times and errors for experimental stimuli (analysis by participants).

|  | **Novel Words**  **(Non-associative condition)** | **Novel Words**  **(Semantic-associative condition)** | **Known Words (Semantic-Associative Condition)** |
| --- | --- | --- | --- |
| **Reaction times (mean)** | 574.70 | 459.03 | 436.39 |
| **Errors (mean)** | 1.13 | 0.45 | 0.40 |
